# Current approaches to incorporation of radium-223 in clinical practice

**DOI:** 10.1038/s41391-017-0020-y

**Published:** 2018-01-03

**Authors:** Chris Parker, Axel Heidenreich, Sten Nilsson, Neal Shore

**Affiliations:** 1 The Institute of Cancer Research, London, UK; 2 The Royal Marsden NHS Foundation Trust, London, UK; 3 University Hospital of Cologne, Cologne, Germany; 4 Karolinska Institute, Stockholm, Sweden; 5 Carolina Urologic Research Center, Myrtle Beach, SC USA

## Abstract

**Background:**

Treatment options for metastatic castration-resistant prostate cancer (mCRPC) have expanded in recent years and include cytotoxic agents (e.g., docetaxel and cabazitaxel), immunotherapy (e.g., sipuleucel-T), oral hormonal therapies targeting the androgen receptor axis (e.g., enzalutamide and abiraterone), and targeted alpha therapy (e.g., radium-223 dichloride (radium-223)). Although treatment guidelines have been updated to reflect the availability of new agents, it is not easy to apply them in daily clinical practice because recommendations vary depending on patient comorbidities and disease characteristics. Furthermore, therapeutic accessibility, clinical judgment, and experience affect the selection of treatment options.

**Methods:**

In this review, we provide practical guidance for the integration of radium-223 into the management of patients with mCRPC based on our collective clinical experience, as well as the available clinical trial data.

**Results:**

Radium-223 is a targeted alpha therapy; as a bone-seeking calcium mimetic, it accumulates in hydroxyapatite areas surrounding tumor lesions and selectively binds to the areas of increased bone turnover. Radium-223 prolongs overall survival and delays time to the first symptomatic skeletal events in men with mCRPC, and is indicated for the treatment of patients with CRPC, symptomatic bone metastases, and no known visceral metastases. We review its clinical efficacy and safety, practical guidance on identifying the appropriate patient, and recommendations for how best to educate and inform prospective patients regarding their treatment decision making. In addition, we review recent evidence for sequential and combination therapies with radium-223, provide our experiences with these treatment approaches, and discuss their implications for the future treatment of patients with mCRPC.

**Conclusions:**

Based on our clinical experience, radium-223 should be considered relatively early in the treatment course in patients with mCRPC with bone metastases. Coordination of care among multidisciplinary team members, patients, and caregivers is essential for optimizing safe and effective treatment with all CRPC therapies.

## Introduction

Metastatic castration-resistant prostate cancer (mCRPC) has been shown to predominantly metastasize to bone and frequently spread to visceral organs and soft tissue as well. Bone metastases occur in up to 90% of patients with mCRPC [1–3], and may be associated with significant clinical complications, including pain [4]; skeletal-related events (SREs), such as pathologic fractures and spinal cord compression [5]; and reduced patient mobility, with quality-of-life (QoL) impairment [4]. Furthermore, the presence of bone metastases is associated with reduced overall survival (OS) [6, 7]. Visceral metastases are less common, but have been observed in ~10% [8] of newly diagnosed patients with mCRPC, increase over time and may affect as many as 49% of men with prostate cancer–specific mortality [1, 3].

Over the last several years, treatment options for mCRPC have expanded and now include cytotoxic agents (e.g., docetaxel and cabazitaxel), oral hormonal therapies targeting the androgen receptor axis (e.g., enzalutamide and abiraterone), targeted alpha therapy (radium-223), immunotherapy (e.g., sipuleucel-T), and bone supportive agents that target bone resorption and reduce SREs (e.g., denosumab and zoledronic acid) [9, 10]. There are several treatment guidelines for mCRPC, including those from the American Urological Association, European Association of Urology, National Comprehensive Cancer Network, Canadian Urological Association, and European Society for Medical Oncology [10–15]. However, despite the abundance of guidelines, it is not always obvious how to best apply them in daily clinical practice when making decisions concerning sequencing and/or combination strategies because recommendations vary depending on patient comorbidities and specific patient disease characteristics. The guidelines typically present a range of treatment options at each stage of the patient journey. In addition, the approach to treatment may vary across clinical practice settings, specifically considering the clinical experience of the physician with the varying approved therapies, and thus possible inherent biases, which may affect the multidisciplinary team involved.

Radium-223 is one of several approved therapies for mCRPC that have been shown to improve OS in patients with mCRPC (Table 1) [1, 16–23]. Ideally, a patient should receive all of the approved therapies during their patient journey. However, all of these CRPC therapies were developed in relatively concurrent trials over the last several years, and published prospective data on their sequential and combined use are severely lacking. Thus, the challenge is to determine the optimum sequence or combination of therapies in the absence of level-one evidence that might help improve specific patient outcomes.

**Table 1:** Efficacy of therapies for metastatic castration-resistant prostate cancer

Therapy	Comparator	Improvement in median overall survival (mo) vs comparator	Hazard ratio
Enzalutamide			
Prechemotherapy [68]	Placebo	4.0	0.77
Postchemotherapy [17]	Placebo	4.8	0.631
Radium-223 + best standard of care [18]	Placebo + best standard of care	3.6	0.70
Cabazitaxel [19]	Mitoxantrone	2.4	0.70
Abiraterone			
Prechemotherapy [23]	Placebo	4.4	0.81
Postchemotherapy [69]	Placebo	4.6	0.74
Docetaxel [1]	Mitoxantrone	2.4	0.76
Sipuleucel-T [22]	Placebo	4.1	0.78

## Methods

In this review, we provide guidance regarding the integration of radium-223 into the management of patients with mCRPC based on our collective clinical experience, as well as the available clinical trial data.

## Results

### Overview of clinical trial data

**Fig. 1:**
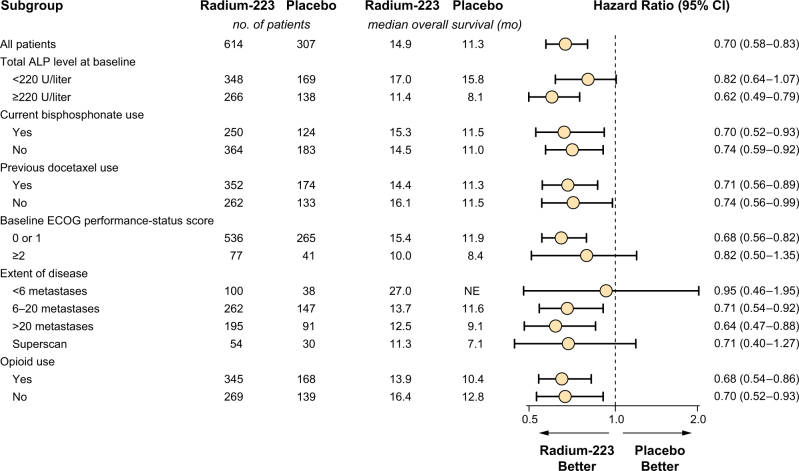
Prospective subgroup analysis of hazard ratios for death in the radium-223 ALSYMPCA trial [18] The Eastern Cooperative Oncology Group (ECOG) scores the performance status of patients with respect to activities of daily living as follows: 0, fully active and able to carry out all predisease activities without restriction; 1, restricted in physically strenuous activity but ambulatory and able to carry out work of a light nature; 2, ambulatory and up and about for more than 50% of waking hours and capable of self-care but unable to carry out work activities; 3, capable of only limited self-care and confined to a bed or chair for more than 50% of waking hours; 4, completely disabled; and 5, dead. The category for use of opioids includes patients with a score of 2 or 3 on the World Health Organization “ladder” for cancer pain (a score of 1 indicates mild pain and no opioid use, 2 indicates moderate pain and occasional opioid use, and 3 indicates severe pain and regular daily opioid use). The category for non-use of opioids includes patients without pain or opioid use at baseline and patients with a score of 1 on the WHO ladder for cancer pain. Superscan refers to a bone scan showing diffuse, intense skeletal uptake of the tracer without renal and background activity. ALP denotes alkaline phosphatase, and NE not evaluated. Reproduced with permission from Parker et al. [18]

Radium-223 was approved based on the results of the international, prospective, randomized, double-blind, placebo-controlled phase 3 ALSYMPCA trial in patients with symptomatic mCRPC and bone metastases. This trial demonstrated that radium-223 plus best standard of care significantly improved OS, leading to a 30% reduction in the risk of death compared with placebo plus best standard of care (Table 2) [18]. Secondary analyses further supported the benefit of radium-223, demonstrating that radium-223 reduced the risk of symptomatic skeletal events (SSEs) and significantly delayed the time to the first SSE [24], was effective regardless of prior docetaxel use (Fig. 1) [25], and provided a significant improvement in patient QoL [26]. Healthcare resource utilization was also an endpoint in the phase 3 study, and a significantly smaller proportion of patients experienced 1 or more hospitalizations with radium-223 than with placebo (37.0% vs 45.5%, respectively; *P* = 0.016) [27]. Exploratory analyses also showed that efficacy (i.e., OS and risk of SSE) was comparable in patients who were symptomatic (baseline opioid use) and minimally symptomatic (no opioid use) [28]. In addition, radium-223 has a well-tolerated safety profile, with a low incidence of myelosuppression regardless of prior docetaxel use [18, 25]. In the phase 3 trial, the most common adverse events (AEs) were bone pain, nausea, and anemia (Table 2) [18]. Ongoing phase 2 and 3 studies with radium-223 are designed to evaluate high-dose and extended treatment regimens, efficacy in combination with the hormonal agents abiraterone and enzalutamide, increased immune response by sipuleucel-T against mCRPC, and treatment effects on biomarkers (ClinicalTrials.gov NCT02043678, NCT02023697, NCT02346526, NCT02034552, NCT02463799, and NCT01929655).

**Table 2:** Key efficacy and safety data for radium-223 from phase 3 trial

Study	Treatment	Patients	Key efficacy results	Key safety findings
Phase 3, randomized, double-blind, placebo-controlled, multicenter ALSYMPCA trial [18]	Ra-223 50 kBq/kg IV or matching placebo q4w × 6	*N* = 921 (Ra-223, *n* = 614; placebo, *n* = 307)	**Primary**	Lower myelosuppression rates and fewer AEs with Ra-223 vs placebo
			Median OS: Ra-223, 14.9 mo; placebo, 11.3 mo; HR, 0.70 (95% CI, 0.58‒0.83; *P* < 0.001) (updated analysis)	
	Patients also received best standard of care	Progressive CRPC with ≥2 bone metastases and no known visceral metastases	**Secondary**	No clinically meaningful differences in frequency of grade 3 or 4 AEs between groups
			Median time to first SSE (Ra-223, 15.6 mo; placebo, 9.8 mo; HR, 0.66 (95% CI, 0.52‒0.83; *P* < 0.001)	
	Patients stratified 2:1 by previous docetaxel use, baseline ALP, and current use vs no use of bisphosphonates	Symptomatic disease with regular use of analgesics or EBRT for painful bone metastases	Time to increase in total ALP level greater with Ra-223 (HR, 0.17 (95% CI, 0.13‒0.22); *P* < *P* value and number together on the same line"?>0.001)	Most common AEs in the Ra-223 group were bone pain (50%), nausea (36%), and anemia (31%)
			Time to increase in PSA level greater with Ra-223 (HR, 0.64 (95% CI, 0.54‒0.77); *P* < 0.001)	

Abbreviations: *AE* adverse event, *ALP* alkaline phosphatase, *ALSYMPCA* Alpharadin in Symptomatic Prostate Cancer, *CRPC* castration-resistant prostate cancer, *EBRT* external beam radiation therapy, *HR* hazard ratio, *IV* intravenous, *kBq* kilobecquerel, *OS* overall survival, *PSA* prostate-specific antigen, *q4w* every 4 wk, *Ra-223* radium-223 dichloride, *SSE* symptomatic skeletal event

### Best clinical practices for treating patients with radium-223

#### Treatment with radium-223

Practice guidelines for radium-223 use vary but generally recommend radium-223 for patients with mCRPC with symptomatic bone metastases [10, 12, 14, 15, 29, 30]. Based on our clinical experience and patient preferences, we generally recommend the selection of less toxic treatments first in an effort to minimize the effect of treatment on patient lifestyle and QoL and to preserve patient daily function (i.e., performance status). Although subgroup analysis of the phase 3 trial suggested that the efficacy of radium-223 is similar across all subgroups examined, radium-223 is particularly appropriate for patients with bone-only or bone-predominant disease.

Although the optimum sequence of life-prolonging therapies is not known, there are several arguments for using radium-223 relatively early in the course of mCRPC, when there is a clinical window of opportunity before the development of visceral metastases. In particular, the probability of developing visceral metastases increases over time, which then renders the patient ineligible for radium-223 per the approved indication [3].

#### Radium-223 in symptomatic patients

The main purpose of treatment with radium-223 is to improve survival, not to relieve symptoms. Thus, symptom severity should not be used as an indication for radium-223 treatment initiation [28]. Further, the term ‘symptomatic’ when describing bone metastases is broadly defined, includes patients who are pain-free on simple analgesia, and may have subjective variation [31, 32].

In the phase 3 trial, symptomatic disease was defined as requiring regular use of analgesic medication or recent use of external beam radiation therapy (EBRT) for cancer-related bone pain [18, 28]. Radium-223 showed a similar improvement in OS both in minimally symptomatic (i.e., patients not using opioid analgesics) and more symptomatic patients with mCRPC [18, 28], suggesting that there is no need to delay treatment with radium-223 until symptoms are severe.

In our practices, radium-223 is generally used early in the management of the disease if the patient exhibits symptomatic bone metastases with or without small volume pelvic or retroperitoneal lymph node metastases, with no evidence of visceral metastases, and with good hematologic function. Symptomatic bone metastases are defined either by actual pain validated on a visual analog scale or by the use of pain medication. The use of radium-223 before or after docetaxel is always discussed with the patient. In addition, we base the decision on several biological parameters, such as prostate-specific antigen (PSA) doubling time, extent of extraosseous disease, and time to castration resistance. In the presence of poor prognostic markers, such as PSA doubling time <6 months, response to androgen deprivation therapy <12 months, elevated lactate dehydrogenase, and extensive lymph node metastases, we favor the use of first-line chemotherapy. In the elderly patient, we suggest using screening tests to objectively assess health status (i.e., fit, vulnerable, or frail) and determine who might be an appropriate candidate for which type of therapy [33–35].

#### Concomitant use of radium-223 with other therapies for CRPC

##### External beam radiation therapy

Because radium-223 was shown to be well tolerated in patients treated with best standard of care, which included local EBRT or treatment with glucocorticoids, antihormonals/antiandrogens, ketoconazole, or estrogens [18], patients receiving any of these therapies could potentially receive concomitant radium-223 treatment, if indicated. This is particularly appropriate in those countries where it is not possible to use radium-223 in combination with abiraterone or enzalutamide. In a phase 2 study, in which all patients received EBRT before study drug, there was no significant difference in hematologic toxicity between radium-223 and placebo [36]. A post hoc analysis of patients receiving EBRT and radium-223 from the phase 3 trial demonstrated that concomitant EBRT did not adversely affect the safety profile of radium-223; the incidence of myelosuppression was low, regardless of concomitant use of EBRT [37]. The safety findings from the phase 3 trial, as well as the non-overlapping mechanism of action of radium-223 with other routine treatments for mCRPC, suggest that the use of radium-223 in combination with best standard of care is feasible in practice [18]. However, concomitant treatment of radium-223 with either cytotoxic chemotherapy or other radionuclides was specifically excluded from the phase 3 trial and should not be used in routine practice unless prospective results become available to support this approach.

##### Hormonal (antiandrogen) therapy

A recent open-label phase 2 study evaluating the combination of radium-223 plus abiraterone in patients with CRPC and symptomatic bone disease reported a decrease in bone pain, an increase in QoL measurements, and stability of ECOG scores compared with the screening visit. Overall, a significant majority of patients had either absence of progressive disease or maintenance of stable disease (eRADicAte; NCT02097303) [38]. A similar study is currently underway evaluating enzalutamide with concurrent administration of radium-223 in patients with CRPC and bone metastases (EnzaRadiCate; NCT02507570). Several additional clinical trials are in progress to investigate combination therapy of radium-223 with either abiraterone or enzalutamide. A phase 3 randomized, double-blind study of radium-223 or placebo, each in combination with abiraterone plus prednisone in chemotherapy-naive patients with asymptomatic or mildly symptomatic mCRPC with bone metastases (ERA 223; NCT02043678) was recently prematurely unblinded[39]. The independent data monitoring committee (IDMC) recommended unblinding the trial due to the observation of more fractures and deaths in the combination treatment arm. Unblinded data from the study are currently being analyzed to confirm the preliminary findings of the IDMC. Given these results from ERA 223 trial, our current recommendation is not to combine radium-223 with concomitant abiraterone acetate and prednisone. A phase 3 multicenter, randomized, open-label study comparing enzalutamide with or without radium-223 in patients with asymptomatic or mildly symptomatic mCRPC with bone metastases is ongoing (PEACE III; NCT02194842).

Based on recent retrospective analyses, it seems there is limited benefit to sequential treatment with abiraterone and enzalutamide in terms of reducing PSA or increasing progression-free survival (PFS) [40]. Moreover, several gene mutations known to occur in androgen receptors have been linked to the development of resistance in response to antiandrogen hormonal therapies, including a mutation that may confer resistance to both abiraterone and enzalutamide [41]. Radium-223 or chemotherapy therefore represents a logical alternative treatment following disease progression on either abiraterone or enzalutamide, rather than sequential use of the other hormonal agent. The data from randomized controlled trials on the efficacy of radium-223 following treatment with either abiraterone or enzalutamide are not available. However, given the lack of resistance to alpha emitter therapy reported in the literature, we would not expect prior treatment with either agent to affect the efficacy of radium-223.

##### Chemotherapy

The optimum treatment sequence of radium-223 relative to docetaxel has not been clinically established. In the phase 3 trial, radium-223 was effective regardless of prior docetaxel use [25]. In our practices, radium-223 was primarily used after docetaxel when radium-223 was first approved; more recently, however, radium-223 is typically used before docetaxel. The choice of using docetaxel or radium-223 as the first agent is generally dependent on the presence of visceral metastases or symptomatic bone disease [42]. More specifically, if visceral disease is not present, then it may be better to use radium-223 before chemotherapy because radium-223 is not indicated for patients with visceral disease [43]. Radium-223 was shown to have a low incidence of myelosuppression in the phase 3 trial; however, prior use of docetaxel therapy (odds ratio (OR), 2.16; *P* = 0.035) and decreased hemoglobin (OR, 1.35; *P* = 0.008) and platelet levels (OR, 1.44; *P* = 0.030) were shown to be risk factors for grades 2–4 thrombocytopenia, which occurred in 6% of patients receiving radium-223 [44]. Consequently, patients with these baseline risk factors should be monitored for hematologic toxicities when receiving radium-223 after chemotherapy [44].

The safety and efficacy of concomitant chemotherapy and radium-223 have not been established, and current recommendations are that radium-223 should not be given with chemotherapy because of concerns about myelosuppression [43,45]. Preliminary findings from a phase 1/2 trial regarding the efficacy and safety of radium-223 plus docetaxel vs docetaxel monotherapy in patients with mCRPC found that the combination was safe and that more patients treated with combination therapy had normalized bone alkaline phosphatase (ALP) levels compared with patients treated with docetaxel alone (39% vs 18% had a decrease >80%) [46]. Patients in the combination treatment group also had longer median PFS compared with docetaxel monotherapy (6.2 vs 4.8 months) [47]. However, these findings should be validated in a larger clinical study; the use of docetaxel in combination with radium-223 cannot be recommended at present.

##### Bone-supportive agents

Administration of radium-223 with bone-supportive agents, such as denosumab or bisphosphonates, has shown good safety profile in patients with mCRPC. In a post hoc exploratory analysis, patients receiving radium-223 with concomitant denosumab had longer median OS compared with patients not treated with denosumab (median OS, not available (95% CI, 15–not available) months vs 13 (12–not available) months), and both groups had a similar safety profile. OS was generally similar in patients treated with radium-223 with concomitant bisphosphonates vs those receiving radium-223 without bisphosphonates [48]. In addition, the ALSYMPCA trial showed that patients receiving radium-223 who were receiving bisphosphonates at study entry had a longer time to first SSE (median, 19.6 (95% CI, 16.5–not estimable) months) vs patients not using bisphosphonates at study entry (11.8 (19.3–13.6) months) [24].

#### Monitoring radium-223 efficacy in patients

In the phase 3 study, imaging was not routinely performed to determine response in bone because conventional computed tomography (CT) scan and Tc bone scan are not considered to be reliable methods to ascertain response in bone [18]. Use of the bone scan index to analyze bone scan findings may be more appropriate. Also, CT may be useful to detect disease progression in areas other than bone and a recent retrospective analysis supports the use of imaging by CT after 3 and 6 doses of radium-223 in order to confirm there has been no extraskeletal disease progression [49]. A number of imaging methods, such as C-11/F-18-choline, F-18-FACBC, and Ga-68-PSMA for positron emission tomography (PET) combined with CT (PET/CT) or magnetic resonance imaging (MRI), are currently under investigation to determine their utility for response assessment in prostate cancer and cannot yet be recommended for use in routine clinical practice. Our recent clinical experience with diffusion-weighted MRI appears initially promising in the assessment of response to radium-223 treatment [50]. Recently published findings from a prospective magnetic resonance substudy of a phase 2 trial of olaparib in mCRPC provide some preliminary evidence of the feasibility of whole-body diffusion-weighted imaging for assessment of bone metastases in patients with mCRPC [51]. However, further evaluation and validation of this approach are needed.

Other routine measurements that we use to assess clinical response and disease progression in our clinical practices include ALP and PSA levels. However, it should be noted that although radium-223 improves survival by 30%, it has a relatively modest effect on serum PSA levels. Specifically, ≥30% reductions in PSA blood levels in the phase 3 trial were achieved by only 16% of patients receiving radium-223 plus best standard of care vs 6% for placebo plus best standard of care (*P* < 0.001) [18]. These PSA results are attributed to the mechanism of action of radium-223 which, like immunotherapy, does not target the androgen receptor and has a relatively modest effect on PSA levels [52]. Thus, a rising PSA level should not, on its own, be an indication to stop therapy with radium-223 [53].

Similarly, although radium-223 has been shown to reduce pain in some patients [18, 54], a lack of pain response should not necessarily be regarded as an indication to stop treatment [52].

#### Monitoring radium-223 safety

Because treatment with radium-223 may cause myelosuppression, blood cell count monitoring should be done at baseline and before each injection. Before the first dose of radium-223, the patient’s absolute neutrophil count (ANC) should be ≥1.5 × 10^9^/L and platelet count ≥100 × 10^9^/L. Before subsequent radium-223 doses, ANC should be ≥1 × 10^9^/L and platelet count ≥50 × 10^9^/L. If these values do not recover within 6–8 weeks after the last radium-223 dose, then radium-223 should be discontinued [43,45].

In our clinical experience, non-hematologic AEs with radium-223, such as transient diarrhea, have been uncommon and rarely require intervention. Treatment discontinuations related to toxicities have been rare in our practice. Other reasons for discontinuation of radium-223 therapy before the completion of 6 treatment cycles include evidence of visceral metastases or rapid disease progression that precludes achieving a treatment response [55]. Long-term (up to 3 years) patient follow-up data from the phase 3 trial showed that radium-223 was well tolerated with a low incidence of myelosuppression and no clinical evidence of long-term safety concerns, such as secondary malignancies or bone marrow failure. Although the possibility of such long-term safety concerns occurring beyond 3 years is unknown, the current safety results are encouraging.

#### Additional considerations and precautions in specific clinical situations

Given the very short range of the alpha-particles and very low emission of gamma photons, treatment with radium-223 does not pose a radiation risk to healthcare professionals. Based on available evidence, no contact restrictions are required for reasons of radiation protection. However, it is important to avoid internal contamination with radium-223. During the first week after administration, low levels of radium-223 may be present in blood, urine, and stool. Moreover, as radium-223 targets the areas of active bone turnover, measurable amounts will be found in bone in the weeks after treatment. When taking the normal precautions that are common practice for healthcare professionals (e.g., preventing transmission of blood-borne infections, such as viral hepatitis), the risks for internal contamination are negligible, even during surgical procedures involving bone.

### Discussions with the patient and their caregiver at the start of radium-223 therapy

The healthcare provider has a responsibility to explain to the patient and their caregiver all of the approved options that are available to treat mCRPC, the rationale for selecting radium-223, and what the patient might expect during treatment (Table 3). The patient should also be made aware that they may eventually receive all other available therapies as needed to control their disease and increase survival over the course of treatment (including docetaxel [56], abiraterone [21], enzalutamide [17], cabazitaxel [19], and sipuleucel-T [57]) whether in combination or sequentially [58–60], depending on drug availability and reimbursement limitations. As part of this discussion, it should be noted that although there are no contraindications for concomitant administration of radium-223 with other approved CRPC therapies (such as abiraterone or enzalutamide), the concomitant administration of chemotherapy or other systemic radioisotopes with radium-223 is contraindicated owing to concerns of myelosuppression [43,45].

**Table 3:** Key elements for patient education

Topic	Key points to emphasize
Multidisciplinary team approach	• Explain the role of each member in ensuring optimal patient care and outcomes
Rationale for choosing Ra-223	• Describe mechanism of action
	• Explain survival benefit
	• Review low incidence of adverse events and types of events to expect
Treatment guidelines	• Review place of radium-223 in treatment guidelines
	• ESMO and NCCN recommend radium-223 with highest level of evidence for both first- and second-line therapy
	• Also recommended by AUA and EAU
	• Highest rating by ESMO-MCBS
Treatment course	• Emphasize importance of adhering to treatment for the full course to derive maximum benefit
	• Explain that a rising PSA or lack of pain response is not necessarily an indication of lack of efficacy
	• Radium-223, similar to immunotherapy, does not target the androgen receptor axis and has a relatively modest effect on PSA levels
Concomitant medications	• Safe when combined with ADT and traditional hormonal therapy
	• Safe when administered with EBRT
	• According to preliminary data, not safe when combined with abiraterone acetate and prednisone
	• Chemotherapy contraindicated until further data are available
Radiation safety	• Reassure patients about minimal risk to patient and caregivers
	• Low levels may be present in blood, urine, and mainly stool during first week after treatment and in bone during weeks after treatment
	• Patients and caregivers should use gloves and follow universal precautions when handling bodily fluids and waste
	•Good hygiene practices should be followed for at least 1 week after the last injection
	•Clothing soiled with patient fecal matter should be washed promptly and separately from other clothing
	• Patients who are sexually active should use condoms and their female partners should use a highly effective method of birth control during treatment and for 6 months following completion of treatment if reproductive potential exists
	• Cremation or burial of a body that had been administered radium-223 does not present a significant risk to crematorium personnel or individuals preparing a body for subsequent burial
	• Pregnant women should not handle radium-223 unless wearing gloves and using proper protection (e.g., barrier gowns)
	• Provide and review instructions for radiation safety
Monitoring efficacy and safety	• Explain that conventional measures of response (e.g., reduction in PSA) may be of limited value because of the different mechanism of action of radium-223; thus, ALP and imaging studies may also be used to monitor treatment response and disease progression
	• Safety monitoring based on regular blood counts and adverse events

Abbreviations: *ADT* androgen deprivation therapy, *ALP* alkaline phosphatase, *AUA* American Urological Association, *EAU* European Association of Urology, *EBRT* external beam radiation therapy, *ESMO* European Society for Medical Oncology, *MCBS* magnitude of clinical benefit scale, *NCCN* National Comprehensive Cancer Network, *PSA* prostate-specific antigen.

As a bone-seeking calcium mimetic, radium-223 accumulates in hydroxyapatite areas surrounding tumor lesions where it binds to newly formed bone stroma in osteoblastic metastases [61, 62]. For patients, it can be helpful to explain the similarity between radium and calcium, emphasizing that radium-223 activity is concentrated in the bone, with limited penetration into soft tissues [61, 62]. A discussion of the most frequently experienced AEs associated with radium-223 will prepare the patient for what to expect during treatment (Table 3) [18].

Patients may be wary of an alpha radiation therapy, so it is also important to explain that the radiation risk is minimal for persons they would come into contact with during treatment, such as their family members [63]. The necessary precautions to ensure radiation safety are much less stringent for radium-223 compared with other well-known radionuclide treatments. Whereas, for example, iodine-131 treatment for thyroid cancer demands hospital admission in shielded rooms and stringent restrictions on direct contact with family and caregivers, the precautions after radium-223 are less demanding. Radium-223 can be administered on an outpatient basis, and there are no restrictions regarding contact with other people immediately after treatment [45] because the alpha-particles emitted by radium-223 only travel a fraction of a millimeter within the body [64]. Radium-223 is rapidly cleared from the blood and primarily distributed into bone or excreted into the intestine, with ~63% of the administered radioactivity being excreted from the body within 7 days post injection [45].

Because radium-223 is eliminated through feces, patients and their caregivers should use gloves and follow universal precautions for patient care when handling bodily fluids and waste to avoid contamination. In general, good hygiene practices should be followed while receiving radium-223 and for at least 1 week after the last injection to minimize the risk of radiation exposure from bodily fluids to household members and caregivers.

It is also helpful to inform patients that radium-223 does not target the androgen receptor and has a relatively modest effect on PSA levels [52]. Consequently, the patient should be made aware that the absence of a PSA response with radium-223 treatment does not necessarily imply a lack of efficacy.

Part of the physician-patient discussion should focus on the importance of adhering to treatment. The recommended dose regimen of radium-223 is 55 kilobecquerel (kBq) per kilogram of body weight given by slow intravenous injection over 1 min at 4-week intervals for a total of six injections [43,45]. An analysis of the open-label, single-arm, phase 3b, international expanded access program for patients with bone-predominant mCRPC found that median OS was greater in patients who received 5–6 injections vs those who received 1–4 injections [65]. Similarly, an OS advantage for patients who received 5–6 injections vs those who received 1–4 injections was seen in post hoc subgroup analyses of the US expanded access program and ALSYMPCA [66]. This suggests that 5 or 6 cycles of treatment might be more effective than fewer cycles, although this was not a randomized comparison. Additional clinical data and/or experience may be necessary to determine whether there is an optimal number of cycles. To this end, a randomized clinical trial is ongoing to assess symptomatic skeletal event-free survival and OS for various doses and regimens of radium-223 (NCT02023697).

### Coordination of care and treatment considerations

Optimal management of patients with mCRPC involves working with a multidisciplinary team that could include a medical oncologist, urologist, radiation oncologist, and nuclear medicine physician and nurse [67]. From a practical standpoint, staff will need to be trained and educated on safe handling and administration of radium-223 [52]. In many countries nuclear medicine specialists will administer radium-223 [55], although the role of nuclear medicine physicians may vary among countries. For example, healthcare providers in a community setting or institution may not have direct access to a nuclear medicine specialist or radiation oncologist [55]. The role of oncologists and urologists may also differ depending on the geographical region or institution, so it is important that the treating physician works with their colleagues based on what is best for the patient in their country or facility [67]. Countries vary in their applicable laws, licenses, and rules on handling and administering radioactive substances. For example, US physicians must have Nuclear Regulatory Commission licensure to administer radium-223; therefore, it is invariably administered by nuclear medicine radiologists or radiation oncologists. In Sweden, radium-223 is prescribed by the oncologist and administered by nuclear medicine radiologists. Because of such differences, compliance with national practices for handling radiopharmaceuticals is essential, and the nuclear medicine physician on the team is typically the most qualified to make arrangements.

## Conclusions

Treatment options for patients with mCRPC have expanded in recent years to include radium-223, a targeted alpha therapy. Based on our collective clinical experience, we recommend that radium-223 be considered relatively early in the course of treatment in patients with mCRPC with bone metastases before they develop visceral disease. Specifically, in patients who progress after receiving hormonal therapy, initiation of radium-223 or chemotherapy may be preferable to switching to another hormonal therapy because of the limited efficacy resulting from cross-resistance. This is particularly important because the clinical window of opportunity for radium-223 exists before the development of visceral metastases, which would render the patient ineligible for radium-223 treatment. Initial findings from the ERA 223 phase 3 clinical trial advise against the combination of radium-223 with abiraterone and prednisone. Coordination of care among multidisciplinary team members, patients, and the patients’ caregivers is essential for optimizing the safe and effective treatment of all CRPC therapies.
